# Outbreak of autochthonous dengue in Fano, Pesaro-Urbino Province - Marche region, Italy, September 2024

**DOI:** 10.1007/s15010-025-02476-1

**Published:** 2025-01-30

**Authors:** Luca Santilli, Benedetta Canovari, Maria Balducci, Giovanni Corbelli, Monia Maracci, Antonio Polenta, Ylenia Farinaccio, Francesco Ginevri, Norma Anzalone, Lucia Franca, Lucia Sterza, Francesco Barchiesi

**Affiliations:** 1 Unit of Infection Diseases, San Salvatore Hospital, AST Pesaro Urbino, Pesaro, Italy; 2 Department of Biomedical Sciences and Public Health, UNIVPM, Ancona, Italy

**Keywords:** Outbreak, Dengue fever, Arboviruses

## Abstract

Dengue is the most common arboviral disease globally. It is caused by four distinct but closely related Dengue viruses (DENV-1, -2, -3, and − 4) transmitted through bites of infected Aedes species mosquito vectors. In the last 50 years, incidence has increased 30-fold with increasing geographic expansion to new countries. Here we report the most important autochthonous Dengue epidemic in Italy ever recorded with a total of 86 confirmed cases occurring in September 2024 in Fano. They were caused by DENV-2. They were 61 Dengue fever, 21 Dengue with warning signs and no cases of severe Dengue. Our data underline the importance of considering Dengue not only as imported disease and rapidly testing all those patients with suggestive clinical pictures even if they have no travel history.

**Supplementary Information:**

The online version contains supplementary material available at 10.1007/s15010-025-02476-1.

## Introduction

**Fig. 1:**
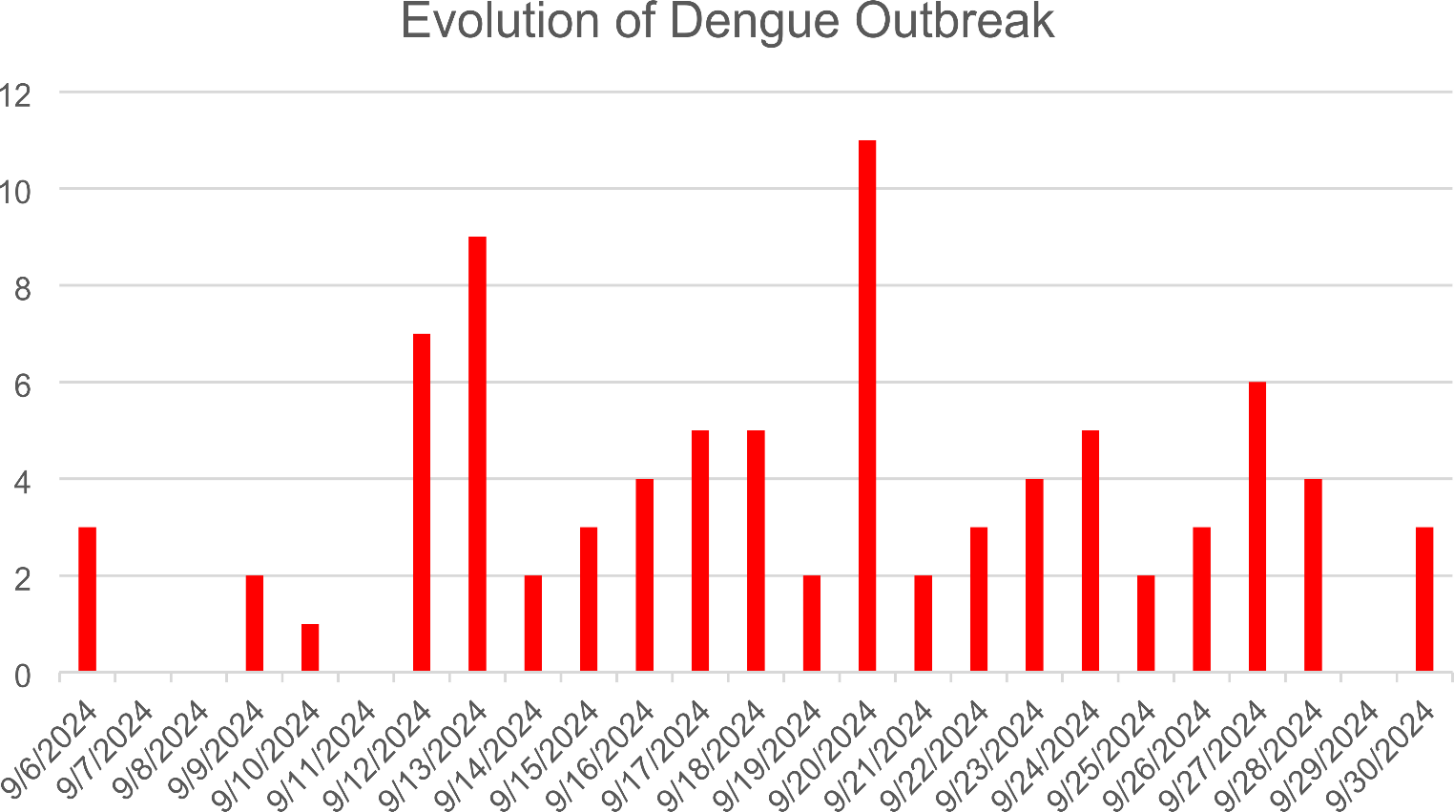
The figure shows new cases per day during Dengue outbreak in the city of Fano from 1st to 30th of September 2024

Dengue fever is a mosquito-borne tropical disease caused by a positive-sense, single-stranded RNA virus belonging to the family of *Flaviviridae*. Dengue is the most common arboviral disease globally. It is caused by four distinct but closely related Dengue viruses (DENV-1, -2, -3, and − 4). DENVs are transmitted through bites of infected *Aedes* species mosquito vectors [1, 2]. In the last 50 years, incidence has increased 30-fold with increasing geographic expansion to new countries [3]. This rapid spread was favoured by various factors such as climate change [4, 5] and globalization which led to a spread of the vector responsible for transmitting the infection, the *Aedes* mosquito. Global incidence of Dengue in 2024 has been the highest on record for this calendar year; many countries are reporting higher-than-usual Dengue case numbers [2]. Dengue is now endemic in all WHO regions except the WHO European Region. Data available for the European region indicate that most cases in the region have been reported by European Union member states, either as incidents in overseas territories or importations from endemic countries [3]. Dengue is characterized by a wide spectrum of clinical manifestations: most cases are asymptomatic or mild and self-managed, presenting fever, nausea, vomiting, rash, aches and pains, and leucopenia. However abdominal pain or tenderness, persistent vomiting, clinical fluid accumulation, mucosal bleeding, lethargy, restlessness, and liver enlargement may occur in a small proportion of cases. These symptoms correlate with severe illness and are characterized by severe plasma leakage leading to shock or fluid accumulation with respiratory distress, severe bleeding, severe organ impairment, impaired consciousness, or cardiovascular disease. Here, we report the most important autochthonous Dengue epidemic in Italy and in Europe ever recorded with a total of 86 confirmed cases (Fig. 1).

## Case description

On the 4th of September a 55-year-old male with fever, arthromyalgia and petechial rash arrived to the Emergency Room of Fano Hospital. Suspecting a viral infection, the patient was sent for a specialist visit to the Infectious Diseases Department of the Pesaro Hospital. No trips abroad were reported in the medical history. Blood tests revealed the presence of thrombocytopenia (PLT 54000/mmc) and hypertransaminasemia (AST 346 U/L and ALT 338 U/L). The patient was discharged with instructions to monitor his blood count values ​​and carry out a series of in-depth tests: Parvovirus B-19, Toscana Virus, Epstein-Barr Virus, Cytomegalovirus, West Nile Virus and Dengue virus. Tests showed: positive Dengue IgM and borderline Dengue IgG, positive Dengue-RNA on blood (serotype 2). On the 7th of September, two women (29 and 37 y.o.) with fever, arthromyalgia and gastrointestinal symptoms (vomiting and diarrhoea) arrived at the emergency room of the Fano hospital. They presented the following blood chemistry tests: thrombocytopenia (32000/mmc and 53000/mmc), severe neutropenia (210/mmc and 800/mmc). The first patient presented also hypertransaminasemia (AST 533 U/L and ALT 335 U/L). They were admitted to the emergency medicine department. Both were tested for the following viruses: Parvovirus B-19, West Nile and Dengue virus. The tests of the first patient showed: positive Dengue IgM and IgG, positive Dengue-RNA on blood (serotype 2).

The test of second patient showed: positive Dengue IgM and borderline Dengue IgG, positive Dengue-RNA on blood (serotype 2).

**Table 1:** The table shows the main laboratory tests of the first three cases of Dengue infection. The reference ranges and corresponding units of measurement are shown above. In bold we reported the altered value

	WBC (4000–11000) Pro mm³	PLT (150000–400000) Pro mm³	Neutrophils (2000–7500) Pro mm³	Lymphocytes (1000–4000) Pro mm³	AST (0–50) U/L	ALT (0–50) U/L
CASE 1	**3780**	**54,000**	**1960**	1030	**346**	**338**
CASE 2	**2290**	**32,000**	**210**	1510	**533**	**335**
CASE 3	**1430**	**53,000**	**800**	**400**	**61**	16

**Fig. 2:**
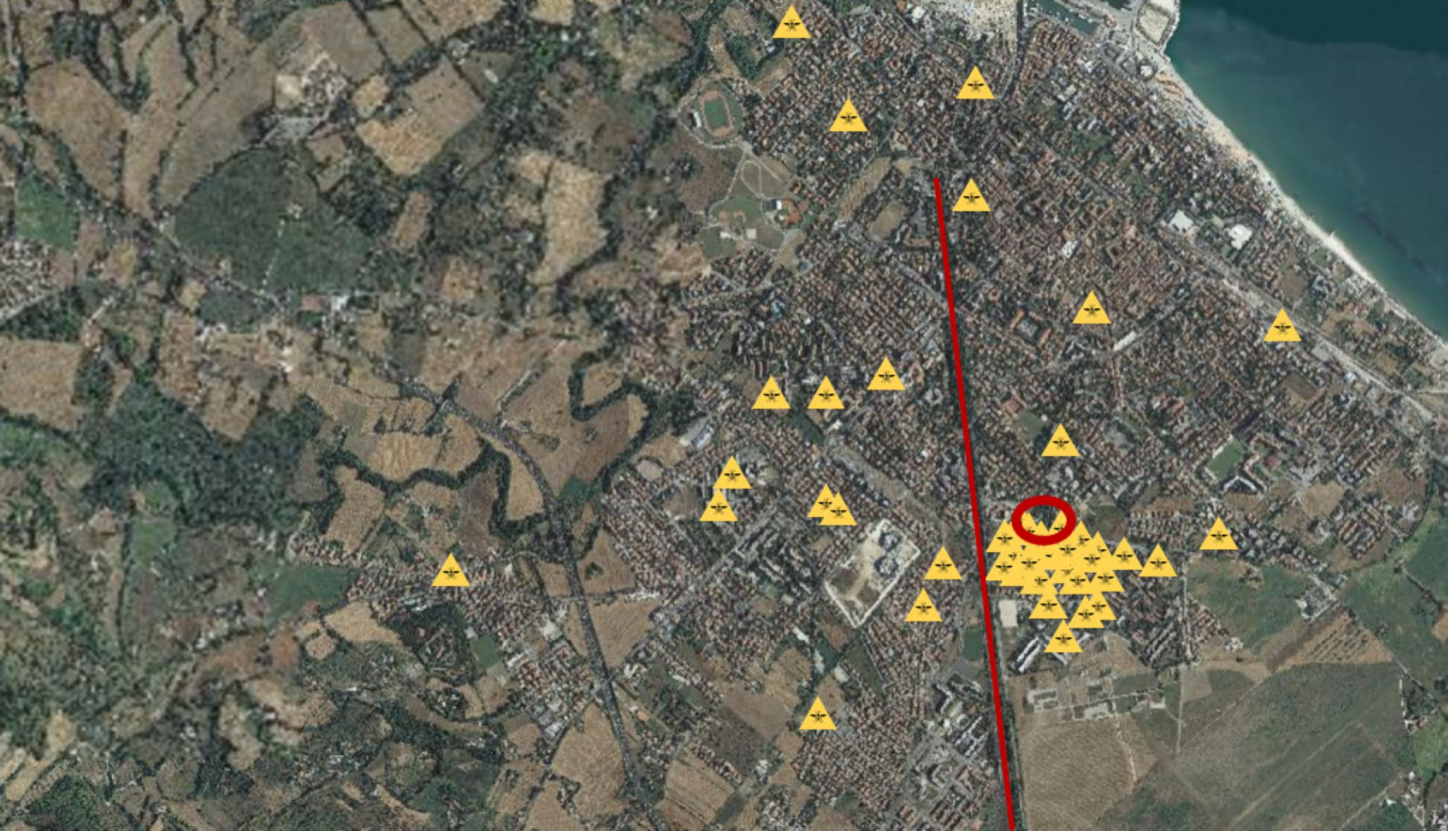
The distribution of cases after one week from the discover of the first Dengue confirmed patient. In red we observe the pond (circle) and the canal (line)

In the following days more and more people accessed the emergency room of the Fano hospital. They all presented a very similar clinical picture with fever, arthromyalgia, rash and gastrointestinal symptoms. Blood tests showed thrombocytopenia, leukopenia and hypertransaminasemia in all cases. Only sporadic cases had a travel history, most of them never moved from Fano. Furthermore, most of the cases were residents in the same area of ​​Fano, close to a pond and a canal with presence of stagnant water (Fig. 2).

On the 30th of September 86 cases of autochthonous Dengue infection serotype 2 were confirmed in the city of Fano with RT-PCR.

Of these 86 patients, 45 (53.5%) were women and 41 (47.7%) men. The median age at diagnosis was 54 y.o. Of these 86 patients, 65 (76%) visited the emergency room. Of these 65, 32 (37.2% of the total) patients required hospitalization. Hospitalized patients received supportive care and close monitoring of blood tests (blood count and liver function). Of the 32 hospitalized patients, only 21 (24.4% of the total) presented a Dengue infection with warning signs. No one presented severe Dengue. No one require transfusion of platelets or blood components. No one were transferred to ICU.

**Fig. 3:**
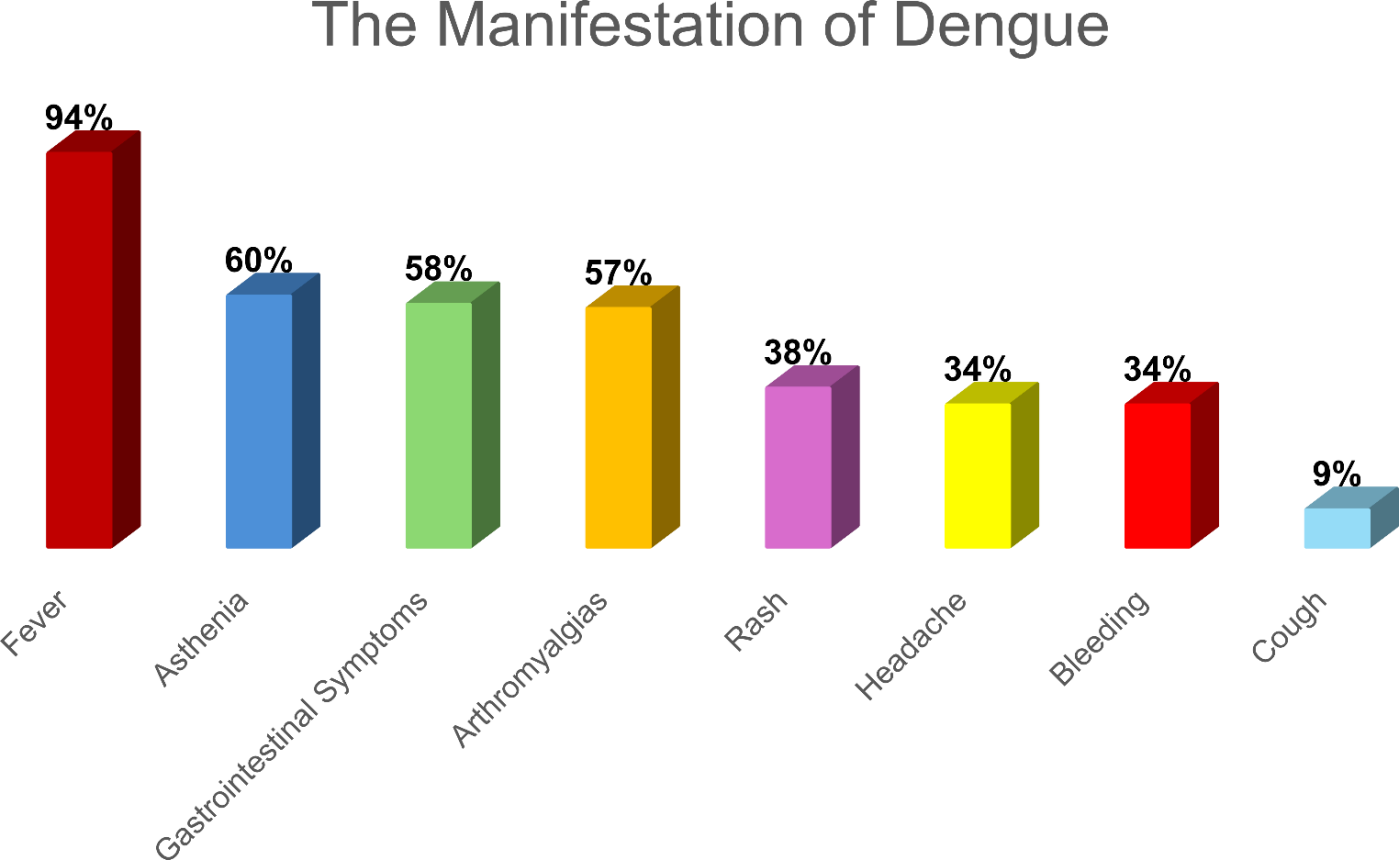
The graphs show the frequency of various symptoms in patients who visited the emergency room for Dengue infection

Among the symptoms most frequently complained by patients there were fever (94% of cases), followed by asthenia (60%), gastrointestinal symptoms (58%) and arthromyalgia (57%). A good percentage also presented bleeding (34%). In some cases, patient presented cough (9%) (Fig. 3).

### Vector control activities and public health measures

The Prevention Department was immediately alerted. An epidemiological investigation was initiated, and all suspected cases were tested for Dengue. The Prevention Department immediately sent a communication to all general practitioners with a recommendation to report all probable cases of Dengue Virus infection and the population was provided with instructions on how to behave in case of suspicious symptoms. We used the case definition criteria of the Italian National Plan for Prevention, Surveillance and Response to Arboviruses (2020–2025) [6]: a probable case is defined as an individual exhibiting symptoms consistent with dengue (fever > 39 °C, nausea or vomiting, rash, aches and pains, retro-ocular pain) with a positive serology for IgM antibodies. A confirmed case requires laboratory confirmation, which may involve virus isolation, detection of viral RNA or Dengue viral antigen (NS1) or the presence of Dengue-specific IgM antibodies in a single serum sample and confirmation by neutralisation or seroconversion or four-fold antibody titre increase of dengue-specific antibodies in paired serum samples. Patients with Dengue fever and no warning signs were managed at home with acetaminophen and oral hydration. Home isolation and the use of mosquito nets and spray repellent to avoid contact with mosquitoes were also recommended. The Prevention Department installed different mosquito traps in the city of Fano in agreement with the Zooprophylactic Institute to conduct typing of the vector insect. Entomological monitoring included BG-sentinel traps (Biogents, Regensburg, Germany) and gravid traps. On 16 and 21 September, three mosquito pools of *Aedes albopictus* tested positive for dengue virus [7]. The city of Fano started an intense disinfestation campaign to control mosquitoes. Adulticidal (cypermethrin-tetramethrin) and larvicide (methoprene pure-denatonium benzoate) disinfectants were used as indicated by the Italian National Plan against arboviral infections [6]. The control measures were taken on 11–12 September within a radius of 200 m from the residence of the index case. As the outbreak spread, a second treatment was scheduled for 15–18 September, targeting the entire municipality of Fano and residential urban areas within a 2 km radius around Fano where cases had been identified. Due to adverse weather conditions on 17–18 September, the treatment was completed on 22–23 September.

The transfusion center of the city of Fano started a massive search for Dengue-RNA on all blood samples and blood products from donors. In all cases, donors were instructed to inform the Blood Establishment in the event of symptoms compatible with DENV infection or diagnosis of DENV infection in the 14 days following the donation (post donation information). No cases of Dengue have emerged among blood donors [8]. On the same date, screening was extended to organs, tissues and haematopoietic stem cell donations across the province. No positive donations were detected.

## Discussion

Since the beginning of 2024, over 12 million dengue cases and over 8000 dengue-related deaths have been reported from 86 countries/territories. Most cases globally have been reported from the WHO PAHO region [9]. In recent years, only sporadic, very small outbreaks of autochthonous Dengue have been reported in Italy [10]: on August 2020 from Veneto region [11, 12]; from August 2023 to October 2023 from Lazio region [13–16] and from Lombardy region [17]. Italy has not been the only European country experiencing autochthonous cases of DENV, other nations, including France, Spain, have also reported instances of autochthonous DENV transmission. We reported the largest outbreak of autochthonous Dengue ever described on Italian territory with a notable impact on the healthcare system of the Province of Pesaro and Urbino. In fact, in September 2024 a significant number of people were hospitalized with a diagnosis of Dengue. The disease is characterized by a wide spectrum of clinical manifestations and often suffers a delay in diagnosis in non-endemic countries [18]. Most Dengue disease cases are asymptomatic or mild and self-managed, presenting fever, nausea, vomiting, rash, aches and pains, and leucopenia. However abdominal pain or tenderness, persistent vomiting, clinical fluid accumulation, mucosal bleeding, lethargy, restlessness, and liver enlargement may occur in a small proportion of cases. These symptoms correlate with severe illness and are characterized by severe plasma leakage leading to shock or fluid accumulation with respiratory distress, severe bleeding, severe organ impairment, impaired consciousness, or cardiovascular disease. During the outbreak, the decision to admit patients with Dengue depended on the emergency room doctor. In most cases, the infectious disease specialist was also consulted. According to WHO Dengue Severity classification, patients with Dengue and warning signs were hospitalized (for example patients with mucosal bleeding or uncontrollable vomiting and at risk of dehydration). In some cases, the presence of low platelets led physician of emergency room to admit patient without warning sings. It’s important to start considering diseases such as Dengue and other arboviruses not only as imported diseases and we have to start testing all patients with suggestive clinical pictures even if they have no travel history. The surge of Dengue cases in Europe has raised significant concerns within the public health domain. Usually associated with tropical climates, the unexpected rise of Dengue infection in these regions underscores the evolving nature of vector-borne diseases and the challenges posed by climate change [19]. Global warming with higher average temperatures, more precipitation and longer periods of drought, changing the distribution of the vectors, could lead to a record-breaking number of Dengue infections worldwide. The impact of climate change on vector distribution, endemic areas, and outbreak frequency is evident for many arboviruses including Dengue, Chikungunya and Zika, tick-borne encephalitis, Crimean-Congo haemorrhagic fever and West Nile virus [20–22]. A One Health approach is vital to prevent the emergence and spread of Dengue to new temperate areas [23, 24]. Another important aspect is the surveillance of arboviruses in travellers. In fact, it is essential to be able to identify a possible arbovirus infection as early as possible in subjects returning from endemic countries. This would allow us to offer the best care and prevent the spread of the disease [25, 26]. Another important aspect to consider is the impact of Dengue virus infection in the elderly population. In fact, older people present a series of comorbidities that can make the course of the infection more severe. In this population we find a greater use of drugs such as antiplatelets and anticoagulants with a greater propensity to bleeding which is already typical of the infection itself. Therefore, it is important to know how to manage polypharmacy in these patients with the possibility of suspending antiplatelet and anticoagulant drugs in cases of severe thrombocytopenia. It is also important to pay attention to signs of dehydration which can lead to a disastrous outcome. A fundamental point is also the control of the main vector of Dengue, the Aedes albopictus mosquito. It is important to intervene with adulticidal and larvicidal disinfestations in a regular and organized manner. Finally, it is important to provide the population with clear and precise information so as not to create panic and avoid overcrowding in the emergency room. In conclusion, we reported the most important autochthonous Dengue epidemic in Italy ever recorded with a total of 86 confirmed cases occurring in September 2024 in Fano Pesaro-Urbino province - Marche region and this event leads us to reconsider the distribution and spread of diseases until now considered exclusive to tropical countries from a broader perspective of climate change and climate tropicalization.

## Electronic supplementary material

Below is the link to the electronic supplementary material.

Supplementary Material 1

## Data Availability

No datasets were generated or analysed during the current study.
