# Supplementary material for: Outbreak of autochthonous dengue in Fano, Pesaro-Urbino Province - Marche region, Italy, September 2024
Source: Infection. 2025 Jan 30;53(3):1213–8. doi: 10.1007/s15010-025-02476-1 (PMC12137368; doi:10.1007/s15010-025-02476-1)
Supplement: Supplementary file 1 — Supplementary Material 1 [file 15010_2025_2476_MOESM1_ESM.docx]

| **AGE** | **DATE ONSET** | **FEVER** | **ARTHROMYALGIA** | **GI SYMPTOMS** | **BLEEDING** | **HEADACHE** | **RASH** | **ASTHENIA** | **COUGH** | **DATE SAMPLE** | **DENGUE IgG** | **DENGUE IgM** | **PCR BLOOD** | **SEROTYPE** |
| --- | --- | --- | --- | --- | --- | --- | --- | --- | --- | --- | --- | --- | --- | --- |
| 55 | 28/08/2024 | + | + | - | + | + | + | + | - | 06/09/2024 | BORDERLINE | + | + | DENV-2 |
| **29** | **30/08/2024** | **+** | **-** | **+** | **-** | **+** | **-** | **+** | **-** | **06/09/2024** | **-** | **+** | **+** | **DENV-2** |
| **34** | **02/09/2024** | **+** | **-** | **+** | **-** | **-** | **-** | **-** | **-** | **09/09/2024** | **-** | **+** | **+** | **DENV-2** |
| **24** | **03/09/2024** | **+** | **-** | **-** | **-** | **+** | **-** | **-** | **-** | **06/09/2024** | **-** | **BORDERLINE** | **+** | **DENV-2** |
| **76** | **03/09/2024** | **-** | **-** | **+** | **-** | **-** | **-** | **+** | **-** | **09/09/2024** | **-** | **+** | **+** | **DENV-2** |
| **37** | **04/09/2024** | **+** | **-** | **-** | **+** | **-** | **-** | **+** | **-** | **10/09/2024** | **+** | **+** | **+** | **DENV-2** |
| **35** | **06/09/2024** | **+** | **+** | **+** | **+** | **+** | **+** | **+** | **-** | **12/09/2024** | **-** | **+** | **+** | **DENV-2** |
| **88** | **09/09/2024** | **+** | **-** | **+** | **+** | **-** | **+** | **+** | **-** | **12/09/2024** | **-** | **BORDERLINE** | **+** | **DENV-2** |
| **40** | **08/09/2024** | **+** | **+** | **-** | **-** | **-** | **+** | **+** | **+** | **12/09/2024** | **+** | **BORDERLINE** | **+** | **DENV-2** |
| **59** | **06/09/2024** | **+** | **-** | **+** | **+** | **+** | **+** | **+** | **-** | **12/09/2024** | **+** | **+** | **+** | **DENV-2** |
| 78 | 13/09/2024 | + | + | + | - | + | + | + | - | 12/09/2024 | + | - | + | DENV-2 |
| 85 | 06/09/2024 | + | - | + | - | - | - | - | - | 13/09/2024 | - | + | + | DENV-2 |
| **48** | **07/09/2024** | **+** | **-** | **+** | **-** | **-** | **-** | **+** | **-** | **13/09/2024** | **-** | **+** | **+** | **DENV-2** |
| 27 | 07/09/2024 | + | - | + | - | - | + | - | - | 13/09/2024 | - | + | + | DENV-2 |
| 78 | 12/09/2024 | - | - | + | - | - | - | - | - | 13/09/2024 | - | + | + | DENV-2 |
| **68** | **29/08/2024** | **+** | **-** | **+** | **+** | **-** | **+** | **-** | **-** | **13/09/2024** | **+** | **+** | **+** | **DENV-2** |
| **46** | **31/08/2024** | **+** | **-** | **-** | **-** | **-** | **+** | **+** | **+** | **13/09/2024** | **+** | **+** | **+** | **DENV-2** |
| 62 | 05/09/2024 | + | + | + | - | + | + | + | + | 13/09/2024 | - | + | + | DENV-2 |
| 45 | 06/09/2024 | + | + | + | - | - | + | + | - | 13/09/2024 | - | + | + | DENV-2 |
| 62 | 05/09/2024 | - | + | - | - | + | + | + | - | 13/09/2024 | BORDERLINE | + | + | DENV-2 |
| 37 | 11/09/2024 | + | + | + | - | - | - | - | - | 14/09/2024 | - | BORDERLINE | + | DENV-2 |
| 73 | 11/09/2024 | + | - | - | - | - | - | - | - | 14/09/2024 | - | - | + | DENV-2 |
| 64 | 14/09/2024 | + | + | - | - | + | - | + | - | 15/09/2024 | - | - | + | DENV-2 |
| **47** | **14/09/2024** | **+** | **-** | **+** | **+** | **+** | **-** | **-** | **-** | **15/09/2024** | **-** | **-** | **+** | **DENV-2** |
| **71** | **14/09/2024** | **+** | **-** | **+** | **-** | **-** | **-** | **+** | **-** | **15/09/2024** | **-** | **-** | **+** | **DENV-2** |
| 20 | 12/09/2024 | + | - | + | - | - | - | - | - | 16/09/2024 | - | + | + | DENV-2 |
| **14** | **11/09/2024** | **+** | **-** | **-** | **+** | **-** | **+** | **+** | **-** | **16/09/2024** | **-** | **+** | **+** | **DENV-2** |
| **82** | **09/09/2024** | **+** | **-** | **-** | **+** | **-** | **-** | **-** | **-** | **16/09/2024** | **-** | **+** | **+** | **DENV-2** |
| **21** | **09//09/2024** | **+** | **-** | **-** | **-** | **-** | **-** | **-** | **+** | **17/09/2024** | **-** | **-** | **+** | **DENV-2** |
| **23** | **12/09/2024** | **+** | **+** | **+** | **+** | **+** | **+** | **+** | **-** | **17/09/2024** | **-** | **+** | **+** | **DENV-2** |
| **73** | **13/09/2024** | **+** | **-** | **+** | **-** | **-** | **-** | **-** | **-** | **17/09/2024** | **-** | **+** | **+** | **DENV-2** |
| 37 | 15/09/2024 | + | + | - | - | - | - | + | - | 17/09/2024 | - | - | + | DENV-2 |
| **68** | **15/09/2024** | **+** | **+** | **+** | **+** | **+** | **+** | **+** | **-** | **18/09/2024** | **-** | **+** | **+** | **DENV-2** |
| **67** | **16/09/2024** | **+** | **+** | **-** | **-** | **-** | **-** | **-** | **+** | **18/09/2024** | **-** | **+** | **+** | **DENV-2** |
| **57** | **18/09/2024** | **+** | **+** | **-** | **-** | **-** | **-** | **-** | **-** | **18/09/2024** | **-** | **+** | **+** | **DENV-2** |
| **2** | **16/09/2024** | **+** | **-** | **+** | **-** | **-** | **+** | **-** | **-** | **18/09/2024** | **-** | **-** | **+** | **DENV-2** |
| **71** | **15/09/2024** | **+** | **-** | **-** | **-** | **-** | **-** | **+** | **-** | **19/09/2024** | **-** | **+** | **+** | **DENV-2** |
| **85** | **17/09/2024** | **+** | **-** | **-** | **-** | **-** | **-** | **-** | **-** | **20/09/2024** | **-** | **-** | **+** | **DENV-2** |
| **73** | **16/09/2024** | **+** | **+** | **-** | **-** | **-** | **-** | **+** | **-** | **20/09/2024** | **+** | **+** | **+** | **DENV-2** |
| 73 | 15/09/2024 | + | + | - | + | - | - | + | - | 20/09/2024 | - | + | + | DENV-2 |
| 35 | 13/09/2024 | + | + | - | + | + | + | + | - | 20/09/2024 | - | + | + | DENV-2 |
| 42 | 20/09/2024 | + | + | - | + | + | + | + | - | 20/09/2024 | BORDERLINE | + | + | DENV-2 |
| 49 | 10/09/2024 | + | + | + | - | - | - | - | - | 21/09/2024 | - | + | + | DENV-2 |
| 49 | 17/09/2024 | + | + | + | - | + | - | + | - | 21/09/2024 | - | - | + | DENV-2 |
| 53 | 15/09/2024 | + | + | - | + | - | + | + | - | 22/09/2024 | - | + | + | DENV-2 |
| 61 | 18/09/2024 | + | + | + | + | - | + | + | - | 22/09/2024 | - | + | + | DENV-2 |
| 54 | 19/09/2024 | + | + | + | - | + | - | + | - | 22/09/2024 | - | + | + | DENV-2 |
| 30 | 18/09/2024 | + | + | - | - | - | - | - | - | 23/09/2024 | - | + | + | DENV-2 |
| 17 | 20/09/2024 | + | + | + | - | - | + | + | - | 23/09/2024 | - | + | + | DENV-2 |
| **80** | **16/09/2024** | **+** | **-** | **-** | **-** | **-** | **-** | **-** | **-** | **23/09/2024** | **-** | **+** | **+** | **DENV-2** |
| 13 | 10/09/2024 | + | + | - | - | + | - | - | - | 23/09/2024 | BORDERLINE | + | + | DENV-2 |
| 64 | 11/09/2024 | + | + | + | + | + | - | + | - | 24/09/2024 | - | + | + | DENV-2 |
| 60 | 16/09/2024 | + | + | - | - | - | + | + | - | 24/09/2024 | - | + | + | DENV-2 |
| **44** | **23/09/2024** | **+** | **-** | **+** | **+** | **-** | **-** | **-** | **-** | **24/09/2024** | **-** | **-** | **+** | **DENV-2** |
| **26** | **20/09/2024** | **+** | **+** | **+** | **+** | **+** | **+** | **+** | **-** | **24/09/2024** | **-** | **+** | **+** | **DENV-2** |
| **75** | **22/09/2024** | **+** | **+** | **+** | **-** | **+** | **-** | **+** | **-** | **25/09/2024** | **-** | **+** | **+** | **DENV-2** |
| **34** | **07/09/2024** | **+** | **+** | **-** | **-** | **-** | **-** | **-** | **-** | **26/09/2024** | **+** | **+** | **+** | **DENV-2** |
| 62 | 09/09/2024 | + | + | + | - | - | - | - | - | 26/09/2024 | + | + | + | DENV-2 |
| 74 | 21/09/2024 | + | + | + | - | - | - | + | - | 27/09/2024 | - | + | + | DENV-2 |
| 30 | 22/09/2024 | + | + | + | - | + | + | + | - | 27/09/2024 | - | + | + | DENV-2 |
| 61 | 27/09/2024 | - | - | + | + | - | - | - | - | 27/09/2024 | - | + | + | DENV-2 |
| 90 | 27/09/2024 | + | + | + | - | - | - | - | - | 28/09/2024 | - | - | + | DENV-2 |
| 24 | 23/09/2024 | + | - | - | + | - | - | + | - | 28/09/2024 | - | + | + | DENV-2 |
| 88 | 27/09/2024 | + | + | + | - | - | - | + | + | 30/09/2024 | - | - | + | DENV-2 |
| **46** | **26/09/2024** | **+** | **+** | **+** | **+** | **+** | **+** | **+** | **-** | **30/09/2024** | **+** | **+** | **+** | **DENV-2** |

*Table 2 The table shows the characteristics of patients who visited the Emergency Room for Dengue infection in September 2024. Patients who have been hospitalized are in bold. The sign “+” means present. The sign “-” means not present.*
